# A novel algorithm for streamlined surgeon-dominated patient-specific implant design in computer-assisted jaw reconstruction

**DOI:** 10.1186/s41205-025-00260-3

**Published:** 2025-03-11

**Authors:** Ankit Nayak, Jane Jingya PU, Xingna YU, Yu-Xiong Su

**Affiliations:** 1 School of Advanced Engineering, UPES, Dehradun, India; 2 Division of Oral and Maxillofacial Surgery, Faculty of Dentistry, The University of Hong Kong, Hong Kong SAR, China

**Keywords:** Computer-assisted surgery, STL, 3D Printing, Jaw reconstruction, Computer-Aided Design (CAD), Bite force correlation, Finite element analysis

## Abstract

**Background:**

Computer-assisted surgery has transformed the approach to jaw resection and reconstruction in recent years. However, the extensive time and technical expertise needed for the planning and creation of patient-specific implants and guides have posed significant challenges for many surgeons in the field. This study introduces a novel algorithm designed to streamline the traditionally intricate and time-consuming Computer-Aided Design (CAD) process for developing patient-specific implants (PSIs).

**Methods:**

The algorithm requires a three-dimensional (3D) model of the reconstructed part. A set of points is selected along the planned location of the plate by the surgeon, defining both the geometry and the positions of the screw holes. These points are then connected to create trace lines, followed by smoothing using cubic-spline data interpolation. Subsequently, a rectangle is swept along the trace line to form the skeleton of the PSI’s surface model. Screw holes are incorporated into the surface model, which is then converted into 3D printable file format. Finite element analysis is conducted to evaluate the functionality of the designed PSI.

**Results:**

Implant design time exhibits significant reductions with the proposed algorithm, which optimizes the model files for printing. Finite Element Analysis is successfully applied to demonstrate the stress levels in the implant plate, which are within safe limits for titanium 3D-printed implants.

**Conclusions:**

This algorithm offers a faster, more efficient, and accurate alternative to traditional CAD methods, with the potential to revolutionize the field of patient-specific implant design. Furthermore, the study demonstrates the utility of a mechanistic model for correlating patient bite force with muscle forces in the literature.

## Introduction

The skeleton of human jaws plays a crucial role in people’s daily life in maintaining the airway for breathing, facial esthetics, mastication, and language articulation. After resection surgeries for pathology involving the upper and lower jaws, it is important to reconstruct the missing structures for patients to resume normal functions [1]. Vascular free tissue transfer from other parts of the body for reconstruction of jaw defects has gained popularity over the years due to its predictable outcome and minimum donor site morbidity [2].

**Fig. 1:**
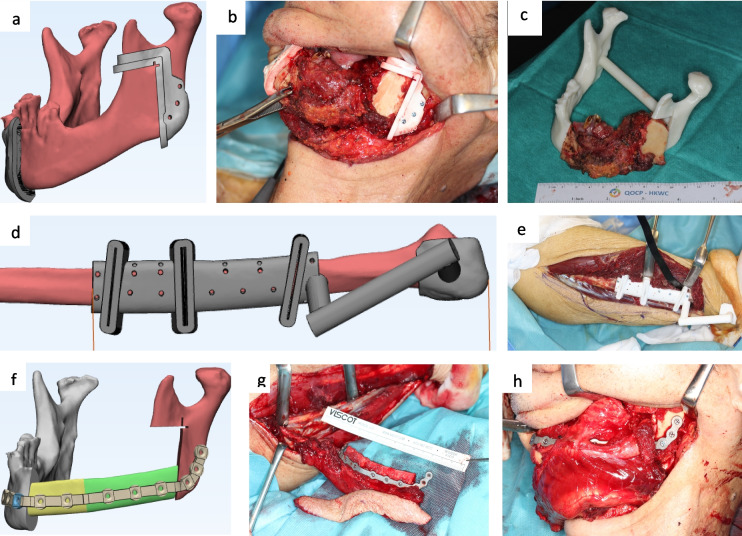
A 72-year-old male patient presented with squamous cell carcinoma of the left lower gingiva who underwent computer-assisted surgery for mandible resection and reconstruction with fibula-free flap. **a** Virtual surgical plan of the resection guides of the mandible. **b** 3D-printed patient-specific guides fixed to the mandible skeleton during the surgery. **c** Resected tumor specimen. **d** Virtual surgical plan of the fibula harvest and segmentation guide. **e** 3D-printed fibula guide fixed to the fibula bone during the surgery. **f** Design of the patient-specific plate. **g** 3D-printed patient-specific plate fixed to the fibula segments to reproduce the shape of mandible. **h** Fibula segments with the plate transferred intraorally to repair the mandible defect

The commonly used donor sites include fibula, iliac crest, and scapula. These bones typically have different shapes from human jaws; however, meticulous trimming is necessary before these bones can be used to reconstruct jaw defects [3]. This process is time-consuming and technique sensitive. This has pushed the development of computer-assisted surgery (CAS). The process has been established and verified in our previous publications [3, 4]. Virtual surgical planning (VSP) of the resection and reconstruction is performed virtually on the computer before the surgery by the surgeons and the engineers. The VSP is transferred to the operating room by the application of 3D-printed patient-specific surgical guides and plates. (Fig. 1) It has greatly facilitated surgeries by making the process safer, more predictable, efficient, and accurate [5, 6]. It has become the new standard of care in many major centres worldwide.

Despite the numerous advantages of CAS, concerns regarding the time spent during the VSP have arisen over the years. As all the guides and plates are patient-specific, they need to be designed case by case before the operation. Reliable planning and designing of the guides requires clinical judgement from surgeons and specific computer-aided design (CAD) skills and several hours of work, which is difficult for surgeons to apply in daily practice. [7–9]

Current PSI design practices use CAD tools to model plates through manual command execution, which can be time-consuming when converting simple 3D geometric shapes into PSIs using Boolean operations [10, 11]. Additionally, sharp corners and edges of manually designed PSIs may cause stress concentration.

This article introduces an innovative method for generating a patient-specific plate solid model. The proposed algorithm produces a smooth design for the patient-specific implant (PSI) to minimize stress concentration at sharp edges and corners. The resulting solid model undergoes finite element analysis (FEA) to evaluate the plate’s strength. This approach aims to streamline the PSI design process, allowing surgeons to create plates more easily and efficiently. While developed for jaw reconstruction, this method can also be generalized for designing plates for other anatomical regions.

## Material and methods

**Fig. 2:**
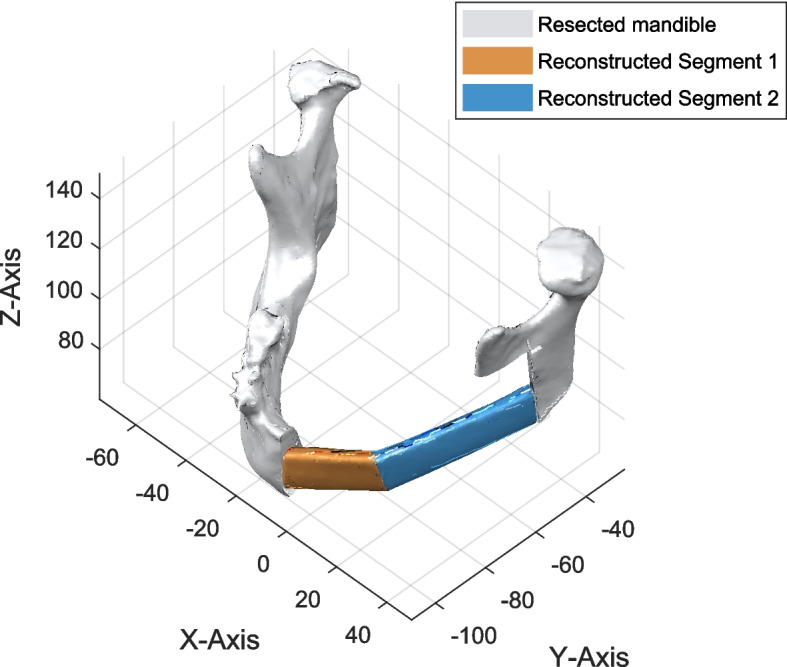
Solid model of the reconstructed mandible

To illustrate the proposed algorithm, we apply it to a reconstructed model of the mandible, as depicted in the Fig. 2. This model is created using a 3D model in STL file format, where the sectioned areas of the mandible have been replaced with fibula flap segments, highlighted in yellow and green in the illustration. The process begins by importing the STL file, which contains the reconstructed bone, into a custom MATLAB program.

The point cloud data of 3D model is represented by an *n* × 3 matrix, where *‘n’* denotes the number of points in the point cloud. The three columns of the matrix correspond to the x, y, and z coordinates of each point in the 3D space.

On the other hand, the connectivity matrix has dimensions of *m* × 3, where ‘*m*’ signifies the number of triangles needed to create the CAD model of the mandible. The three different arrays in the matrix represent the connectivity of the points, so if the three points stored in a row of the connectivity matrix are joined, they will form a triangle.

Upon receiving the STL file of the reconstructed mandible model, two three-dimensional curves are created on its surface (as attached) by selecting and connecting specific points on the 3D model. The surgeon chooses these points to indicate the intended position of the plate. Additionally, another set of points is selected to indicate the locations for the screws that will secure the Patient-Specific Instrument (PSI) to the reconstructed model. The computer program facilitates the visualization of the mandible’s geometry, allowing for interactive definition of the points for guide curves and screw holes.

These points are stored in three different arrays C1, C2 and P1. C1 and C2 are the point data set for two curves and P1 array contains the coordinates of the screw hole location. These matrices stored $n,$ $m$ and $k$ number of points and have $n\times 3$, $m\times 3$ and $k\times 3$ dimensions respectively.

The selection of points is a manual process performed by the surgeon. Various input methods, such as a mouse, digital pen, or touchpad, can be used to select these points. The 3D model of the reconstructed part can be interactively rotated, panned, and zoomed in a developed MATLAB program, facilitating the selection of points on the surface of the 3D model. Defining numerous points to attach curves to the 3D model (STL) can be time-consuming. To enhance automation, cubic-spline data interpolation has been employed to calculate intermediate points between each pair of manually selected points and to fit a spline curve accordingly. This approach minimizes the need to define an extensive number of points for curve attachment.

### Cubic-spline data interpolation

Cubic spline interpolation is a technique often used in research to estimate new data points within a given set of known points. This method involves creating an interpolation function, known as a spline, which is made up of several cubic polynomial segments. The new data points are then calculated as function values of this spline. Cubic-spline data interpolation interpolates the manually defined points and inserts the finite number of points between each pair of the points.

**Fig. 3:**
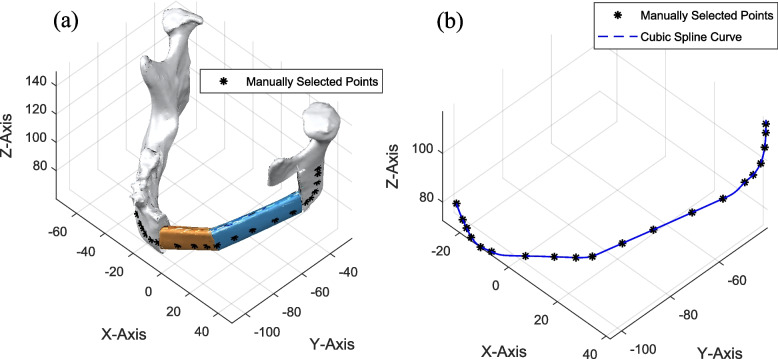
**a** Manually selected data points for curve, **b** curve generated from cubic spline data interpolation

To explain the cubic-spline data interpolation, we only consider the data set of curve 1 that is represented by C1 and has n number of points. Each pair of two consecutive points is interpolated to calculate the *q* number of intermediate points. This forms a cubic spline curve as shown in the Fig. 3.

The curve produced by cubic spline interpolation can exhibit sharp corners and self-intersections, as illustrated in Fig. 3. 3D geometries generated along this curve by sweeping a rectangle may lead to stress concentrations in the plate or inaccuracies in the STL at these corners. To mitigate such sharp points, the curve is further smoothed using the cubic smoothing spline algorithm.

This method fits a smooth curve to a given dataset employing piecewise cubic polynomials and can be executed in MATLAB using the “csaps” function. The algorithm computes a cubic smoothing spline that strikes a balance between the closeness of the data points to the curve and the preservation of the curve’s smoothness. This balance is achieved by minimizing a combined metric that encompasses a weighted sum of the residual sum of squares along with the integral of the squared second derivative of the spline function.

In the present case, spline C1 have n data points and each data point has x y and z coordinates and represents as follows-

$$C1=\left[\begin{array}{c}\begin{array}{ccc}{x}_{1}& {y}_{1}& {z}_{1}\\ {x}_{2}& {y}_{2}& {z}_{2}\\ \vdots & \vdots & \vdots \end{array}\\ \begin{array}{ccc}{x}_{n}& {y}_{n}& {z}_{n}\end{array}\end{array}\right]$$

For the given set of data, the x y and z coordinates of each point are smoothened against a normalized scale ns = [1,2,3,4,….n]. So, the cubic spline smoothing of each column of the C1 matrix is performed against ns, and then smoothen datasets are combined to form the smooth curve. Let us consider the smoothing of the first column $X=\left[{x}_{1},{x}_{2},\dots ,{x}_{n}\right]$ of C1 to explain this process. Points (${ns}_{i}$, ${x}_{i}$) for i = 1,2, …, n, the cubic smoothing spline S(*ns*) is defined as the function that minimizes the following objective function:

1 $$P \sum_{i=1}^{n}{{w}_{i}\left|{x}_{i}- S\left({ns}_{i}\right)\right|}^{2}+ \left(1-P\right)\int {\lambda \left(ns\right)\left[{S}^{{\prime}{\prime}}\left(ns\right)\right]}^{2}dns$$

**Fig. 4:**
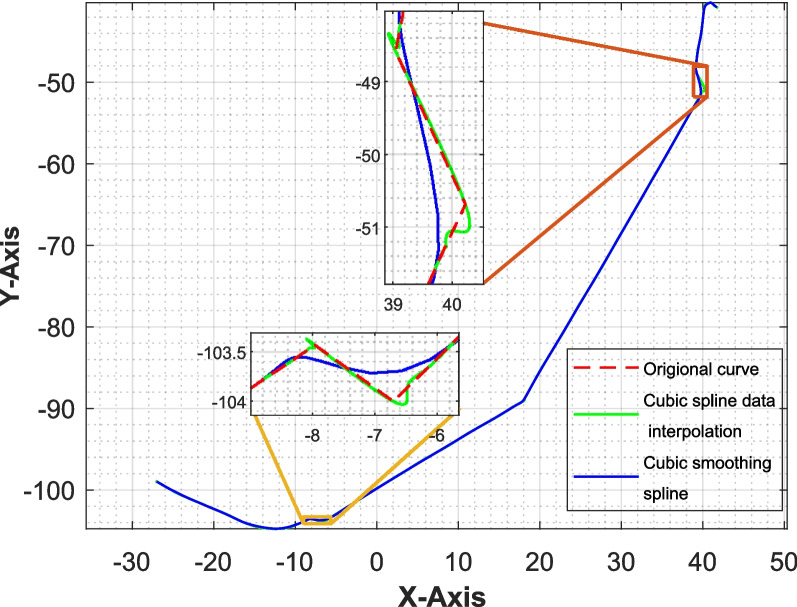
Smoothing of the curve

Here, P is a smoothing parameter that ranges from 0 to 1. The first term in the objective function represents the residual sum of squares, which quantifies the discrepancy between the data points and the spline function. The second term is the integral of the squared second derivative of the spline function, which measures the smoothness of the curve. When P = 1, the algorithm produces a least-squares cubic spline, and when P = 0, it results in a variational spline Fig. 4.

In the “csaps” function, the cubic smoothing spline is constructed using a set of piecewise cubic polynomials defined over adjacent intervals of the data points. These polynomials are continuous and have continuous first and second derivatives, ensuring the smoothness of the spline function. The function takes the input data points (${ns}_{i}$, ${x}_{i}$) and the smoothing parameter P as inputs and returns the coefficients of the cubic polynomials that define the smoothing spline $\overline{X }=\left[{\overline{x} }_{1},{\overline{x} }_{2},\dots ,{\overline{x} }_{n}\right]$.

Similarly, the smooth spline is calculated for each column of C1 i.e. $\overline{Y }=\left[{\overline{y} }_{1},{\overline{y} }_{2},\dots ,{\overline{y} }_{n}\right]$ and $\overline{Z }=\left[{\overline{z} }_{1},{\overline{z} }_{2},\dots ,{\overline{z} }_{n}\right]$. The smoothened spline array of each coordinate then combines to form a *n* × 3 matrix of smoothened 3D curve $$\overline{C1 }=[\begin{array}{ccc}{\overline{X} }^{T}& {\overline{Y} }^{T}& {\overline{Z} }^{T}\end{array}]$$.

In the same way, the second curve C2 is processed and now it become $\overline{C2 }$ and points stored in $\overline{C2 }$ are arranged in a sequence so that the Euclidean distance between i^th^ points of both of the curves should be minimal.

The second curve $\overline{C1 }$ is processed and transformed into a new curve $\overline{C2 }$. The points stored in $\overline{C2 }$ are arranged in a sequence such that the Euclidean distance between the i^th^ points of both curves, $\overline{C1 }$ and $\overline{C2 }$, is minimized. The smoothened curves can be represented as-

$$\overline{C1}=\begin{bmatrix}\begin{array}{ccc}{\overline x}_1&{\overline y}_1&{\overline z}_1\\{\overline x}_2&{\overline y}_2&{\overline z}_2\\\vdots&\vdots&\vdots\end{array}\\\begin{array}{ccc}{\overline x}_n&{\overline y}_n&{\overline z}_n\end{array}\end{bmatrix}\;\mathrm{and}\;\overline{C2}=\begin{bmatrix}\begin{array}{ccc}{\overline k}_1&{\overline l}_1&{\overline m}_1\\{\overline k}_2&{\overline l}_2&{\overline m}_2\\\vdots&\vdots&\vdots\end{array}\\\begin{array}{ccc}{\overline k}_n&{\overline l}_n&{\overline m}_n\end{array}\end{bmatrix}$$

### Sweep the rectangle

The dimensions of the rectangle are determined by the plate’s thickness and width, and these dimensions can be adjusted by the user. In this study, the rectangle’s height is set at 4.62 mm, and its width is set at 2 mm.

**Fig. 5:**
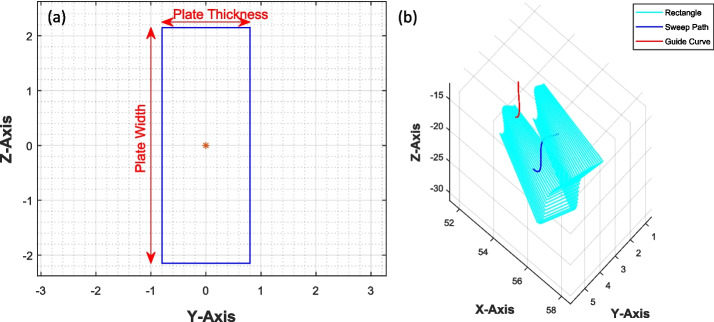
**a** Rectangle representing the cross-sectional area of the surgical plate. **b** Geometry (surface model of the surgical plate) is generated by sweeping the rectangle along the sweep path and guide curve

The generated curves C1 and C2 function as the sweep path and guiding curve, respectively. To reduce the complexity of transformation-related calculations, the rectangle is centered at the origin, as shown in Fig. 5(a). The rectangle is then swept along curve C1 while simultaneously being tilted about its center to conform to the curvature of C2, as illustrated in Fig. 5(b). The surface model of the surgical plate is derived from the data points acquired by sweeping the rectangle along curve C1 and the guiding curve C2.

To follow the curvature of the sweep path and the guide curve the two conditions are necessary to consider. 1) Maintain the normal to the polygon in the instantaneous direction of the tangent to the path. 2) Rotate the rectangle about its normal so that the longest side of the rectangle becomes parallel to the angulation vector $\overrightarrow{\overline{C1 }-\overline{C2} }$.

From the sweep path and guide curve the instantaneous vector ($\widehat{v})$ and angulation vector ($\widehat{u})$ are calculated for each point of the sweep path (Eq. 2aa and 2bb).

2a $$\overrightarrow{{v}_{n}}={c}_{n}-{c}_{n-1}$$

2b $$\overrightarrow{{u}_{n}}={b}_{n}-{c}_{n}$$

where,

$${c}_{n}=\left[\begin{array}{ccc}{\overline{x} }_{n}& {\overline{y} }_{n}& {\overline{z} }_{n}\end{array}\right]$$

$${b}_{n}=\left[\begin{array}{ccc}{\overline{k} }_{n}& {\overline{l} }_{n}& {\overline{m} }_{n}\end{array}\right]$$

3 $${\widehat{v}}_{n}=\frac{{\overrightarrow{v}}_{n}}{\Vert {\overrightarrow{v}}_{n}\Vert }$$

4 $${\widehat{u}}_{n}=\frac{\left[{\overrightarrow{u}}_{n}\right]}{\Vert {\overrightarrow{u}}_{n}\Vert }$$

To align the normal of the rectangle [G] with the instantaneous tangent of the curve the inclination $$\left[\begin{array}{cc}{\alpha }_{n}& {\beta }_{n}\end{array}\right]$$ of each instantaneous tangent vector ($\widehat{v})$ with the 2-principal axis $$\widehat{x}=\left[\begin{array}{ccc}1& 0& 0\end{array}\right]$$ and $$\widehat{y}=\left[\begin{array}{ccc}0& 1& 0\end{array}\right]$$ are calculated and the rectangle coordinates were transformed by multiplying the rotation matrix Rz and Ry sequentially. Rz represents the rotation matrix about the z-axis while Ry is the rotation matrix about the updated axis ${\overrightarrow{A}}_{y}$ of the rectangle after rotation about the z axis.

In order to align the normal of the rectangle [G] with the instantaneous tangent of the curve, the inclination $$\left[\begin{array}{cc}{\alpha }_{n}& {\beta }_{n}\end{array}\right]$$ of each instantaneous tangent vector ($\widehat{v})$ with respect to the 2-principal axis $$\widehat{x}=\left[\begin{array}{ccc}1& 0& 0\end{array}\right]$$ and $$\widehat{y}=\left[\begin{array}{ccc}0& 1& 0\end{array}\right]$$ was calculated, and the rectangle coordinates were transformed by sequentially multiplying it with the rotation matrices Rz and Ry. Rz represents the rotation matrix about the z-axis, while Ry represents the rotation matrix about the updated rotational axis Ay of the rectangle after rotation about the z-axis.

5 $$\left[G\right]\left[{R}_{z}\right]=\left[\begin{array}{c}\begin{array}{ccc}{-g}_{11}\text{sin}{\alpha }_{n}& {g}_{11}\text{cos}{\alpha }_{n}& \begin{array}{cc}{g}_{12}& 1\end{array}\end{array}\\ \begin{array}{ccc}{-g}_{21}\text{sin}{\alpha }_{n}& {g}_{21}\text{cos}{\alpha }_{n}& \begin{array}{cc}{g}_{22}& 1\end{array}\end{array}\\ \begin{array}{c}\begin{array}{ccc}{-g}_{31}\text{sin}{\alpha }_{n}& {g}_{31}\text{cos}{\alpha }_{n}& \begin{array}{cc}{g}_{32}& 1\end{array}\end{array}\\ \begin{array}{ccc}{-g}_{41}\text{sin}{\alpha }_{n}& {g}_{41}\text{cos}{\alpha }_{n}& \begin{array}{cc}{g}_{42}& 1\end{array}\end{array}\end{array}\end{array}\right]$$

From this matrix, the updated rotational axis A_y_ can be calculated as

6 $${\overrightarrow{A}}_{y}=\frac{1}{2}\left[\begin{array}{c}-\left({g}_{11}+{g}_{21}{-g}_{31}-{g}_{41}\right)\text{sin}{\alpha }_{n}\\ \left({g}_{11}+{g}_{21}{-g}_{31}-{g}_{41}\right)\text{cos}{\alpha }_{n}\\ \left({g}_{12}+{g}_{22}{-g}_{32}-{g}_{42}\right)\end{array}\right]$$

This can be converted into a unit vector.

7 $${\widehat{A}}_{y}=\frac{{\overrightarrow{A}}_{y}}{\Vert {\overrightarrow{A}}_{y}\Vert }=\left[\begin{array}{c}{a}_{x}\\ {a}_{y}\\ {a}_{z}\end{array}\right]$$

Upon determining the unit vector of rotation using Eq. 7, the transformation matrix Ry is computed according to Eq. 8. Subsequently, this matrix is employed to transform the point cloud of the rectangle, which has been previously calculated via Eq. 5. Through this multiplication, the rectangle’s normal vector is effectively aligned with the instantaneous tangent of the curve. Moreover, the rectangle points are translated to their corresponding coordinates on the sweep path. The translation matrix, denoted as T_r_, is illustrated in Eq. 9. This alignment operation is critical for ensuring the accurate representation of the geometric properties of the reconstructed part, which, in turn, is essential for the precise sweeping operation of the rectangle along the sweep path.

8 $$\left[{R}_{y}\right]=\left[\begin{array}{ccc}\begin{array}{c}\text{cos}{\beta }_{n}+{a}_{x}^{2}(1-\text{cos}{\beta }_{n})\\ {a}_{x}{a}_{y}\left(1-\text{cos}{\beta }_{n}\right)+{a}_{z}\text{sin}{\beta }_{n}\\ \begin{array}{c}{a}_{z}{a}_{x}\left(1-\text{cos}{\beta }_{n}\right)-{a}_{y}\text{sin}{\beta }_{n}\\ 0\end{array}\end{array}& \begin{array}{c}{a}_{x}{a}_{y}\left(1-\text{cos}{\beta }_{n}\right)-{a}_{z}\text{sin}{\beta }_{n}\\ \text{cos}{\beta }_{n}+{a}_{y}^{2}(1-\text{cos}{\beta }_{n})\\ \begin{array}{c}{a}_{z}{a}_{y}\left(1-\text{cos}{\beta }_{n}\right)-{a}_{x}\text{sin}{\beta }_{n}\\ 0\end{array}\end{array}& \begin{array}{cc}\begin{array}{c}{a}_{x}{a}_{z}\left(1-\text{cos}{\beta }_{n}\right)+{a}_{y}\text{sin}{\beta }_{n}\\ {a}_{y}{a}_{z}\left(1-\text{cos}{\beta }_{n}\right)-{a}_{y}\text{sin}{\beta }_{n}\\ \begin{array}{c}\text{cos}{\beta }_{n}+{a}_{z}^{2}(1-\text{cos}{\beta }_{n})\\ 0\end{array}\end{array}& \begin{array}{c}0\\ 0\\ \begin{array}{c}0\\ 1\end{array}\end{array}\end{array}\end{array}\right]$$

9 $$\left[{T}_{r}\right]=\left[\begin{array}{cc}\begin{array}{cc}\begin{array}{c}\begin{array}{c}1\\ 0\end{array}\\ \begin{array}{c}0\\ {\overline{x} }_{n}\end{array}\end{array}& \begin{array}{c}\begin{array}{c}0\\ 1\end{array}\\ \begin{array}{c}0\\ {\overline{y} }_{n}\end{array}\end{array}\end{array}& \begin{array}{cc}\begin{array}{c}\begin{array}{c}0\\ 0\end{array}\\ \begin{array}{c}1\\ {\overline{z} }_{n}\end{array}\end{array}& \begin{array}{c}\begin{array}{c}0\\ 0\end{array}\\ \begin{array}{c}0\\ 1\end{array}\end{array}\end{array}\end{array}\right]$$

10 $$\left[{G}{\prime}\right]=\left[G\right]\left[{R}_{z}\right]\left[{R}_{y}\right]\left[{T}_{r}\right]$$

Matrix G’ represents the transformed coordinates of the rectangle. The first, second, and third columns of the matrix display the x, y, and z coordinates of the four points of the rectangle, respectively. This can be expressed as shown in Eq. 11.

11 $$\left[{G}{\prime}\right]=\left[\begin{array}{cc}\begin{array}{cc}\begin{array}{c}\begin{array}{c}{g}_{11}{\prime}\\ {g}_{21}{\prime}\end{array}\\ \begin{array}{c}{g}_{31}{\prime}\\ {g}_{41}{\prime}\end{array}\end{array}& \begin{array}{c}\begin{array}{c}{g}_{12}{\prime}\\ {g}_{22}{\prime}\end{array}\\ \begin{array}{c}{g}_{32}{\prime}\\ {g}_{42}{\prime}\end{array}\end{array}\end{array}& \begin{array}{cc}\begin{array}{c}\begin{array}{c}{g}_{13}{\prime}\\ {g}_{23}{\prime}\end{array}\\ \begin{array}{c}{g}_{33}{\prime}\\ {g}_{43}{\prime}\end{array}\end{array}& \begin{array}{c}\begin{array}{c}1\\ 1\end{array}\\ \begin{array}{c}1\\ 1\end{array}\end{array}\end{array}\end{array}\right]$$

From this array the alignment vector ${w}_{n}$ of rectangle is calculated

12 $$\left[{w}_{n}\right]=\left[{\frac{1}{2}(g}_{1}{\prime}+{g}_{4}{\prime})\right]-{c}_{n}$$

Now, the angle ${\gamma }_{n}$ between vectors ${\overrightarrow{w}}_{n}$ and ${\overrightarrow{u}}_{n}$ is computed, followed by the rotation of the rectangle by ${\gamma }_{n}$ with respect to its normal vector ${\widehat{v}}_{n}$. This adjustment ensures that the inclination of each rectangle aligns the width of the plane with the surface of the reconstructed mandible, thereby maintaining accuracy of the plate structure according to the reconstructed part of the mandible.

### Placement of the hole

The surgical plate is designed with multiple through holes that allow for secure fixation to the mandible using screw fasteners. The surgeon determines the precise placement of these fasteners in relation to the corresponding holes. Additionally, holes are provided at both ends of the plate.

These holes are positioned at the center of the plate’s width. The developed computer program allows users to specify the hole points prior to defining curve C1.

13 $$\begin{array}{c}\left[H\right]=\begin{bmatrix}h_1&h2&...&h_n\end{bmatrix}\\H\underline\subset\overline{C1}\end{array}$$

*Where*

*h*_*1*_* and h*_*n*_ = *holes at the ends*

*h*_*2*_* to h*_*n-1*_ = *Manually defied holes*

The screw holes in the plate create a narrow margin, which may result in stress concentration and ultimately lead to fractures in the plate. To mitigate this issue, an additional margin around the screw holes is included, which could effectively manage the stress distribution throughout the plate.

To expand the plate’s width around the screw holes, the rectangles adjacent to the holes are stretched along the z-axis (refer to Fig. 5(a)), and their height is increased proportionally to the distance between the rectangle’s centre point and the nearest hole’s centre, as illustrated in Eq. 14. This distance is represented by D, denoting the diameter of the circular structure surrounding the hole with diameter d.

**Fig. 6:**
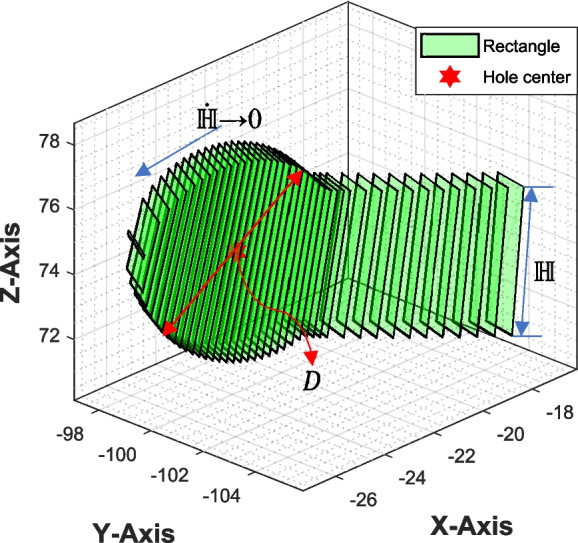
Rectangles have a variable height at the end to generate a circular disc-shaped structure

Furthermore, at the ends of the plate, the height of the rectangle conforms to the equation of a circle, creating a disk-shaped structure as illustrated in the Fig. 6.

14 $${\mathbb{H}}_{n}=\left\{\begin{array}{c}H, k>D/2\\ \dot{\mathbb{H}}, k\le D/2\end{array}\right.$$

15 $$\dot{\mathbb{H}}=\frac{D}{2}\text{sin}\left\{{\text{cos}}^{-1}\left(\frac{k}{D/2}\right)\right\}$$

where,

k = distance of ${c}_{n}$ from the centre of the nearest hole

${\mathbb{H}}=$ half of the plate width

### Elimination of intersecting rectangles

**Fig. 7:**
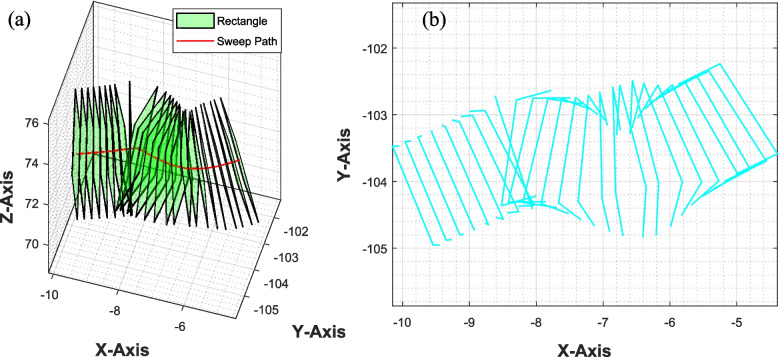
**a** Intersecting rectangles along the sweep path, **b** top view of the rectangles showing intersection in the block of corners (−9, −105) and (−8, −104)

After applying cubic spline smoothing to the sweep path, we can ensure a smooth finish for the plate. However, in instances of complemented geometry, we have observed some intersections between a few rectangles, as illustrated in the Fig. 7. Fortunately, these intersecting rectangles are relatively uncommon, so the algorithm includes a function that detects and excludes them from the plate’s surface model.

The intersection detection algorithm (function) efficiently identifies the intersection between two 3D rectangular regions (${G}_{n}^\prime$, ${G}_{(n+1)}^\prime$). The primary input to this function consists of two 4 × 3 matrices, each representing a 3D rectangle through the (x, y, z) coordinates of its four corners represented by Eq. 15 and 16. The algorithm initiates the process by calculating the edges ${\overrightarrow{E}}_{n}$ and ${\overrightarrow{E}}_{(n+1)}$ of both input rectangles, as shown in Eqs. 17 and 18.

16 $$\left[{G}_{n}^\prime\right]=\left[\begin{array}{cc}\begin{array}{cc}\begin{array}{c}\begin{array}{c}{{g}_{n}{\prime}}_{11}\\ {{g}_{n}{\prime}}_{21}\end{array}\\ \begin{array}{c}{{g}_{n}{\prime}}_{31}\\ {{g}_{n}{\prime}}_{41}\end{array}\end{array}& \begin{array}{c}\begin{array}{c}{{g}_{n}{\prime}}_{12}\\ {{g}_{n}{\prime}}_{22}\end{array}\\ \begin{array}{c}{{g}_{n}{\prime}}_{32}\\ {{g}_{n}{\prime}}_{42}\end{array}\end{array}\end{array}& \begin{array}{c}\begin{array}{c}{{g}_{n}{\prime}}_{13}\\ {{g}_{n}{\prime}}_{23}\end{array}\\ \begin{array}{c}{{g}_{n}{\prime}}_{33}\\ {{g}_{n}{\prime}}_{43}\end{array}\end{array}\end{array}\right]$$

17 $$\left[{G}_{(n+1)}^\prime\right]=\left[\begin{array}{cc}\begin{array}{cc}\begin{array}{c}\begin{array}{c}{{g}_{(n+1)}{\prime}}_{11}\\ {{g}_{(n+1)}{\prime}}_{21}\end{array}\\ \begin{array}{c}{{g}_{(n+1)}{\prime}}_{31}\\ {{g}_{(n+1)}{\prime}}_{41}\end{array}\end{array}& \begin{array}{c}\begin{array}{c}{{g}_{(n+1)}{\prime}}_{12}\\ {{g}_{(n+1)}{\prime}}_{22}\end{array}\\ \begin{array}{c}{{g}_{(n+1)}{\prime}}_{32}\\ {{g}_{(n+1)}{\prime}}_{42}\end{array}\end{array}\end{array}& \begin{array}{c}\begin{array}{c}{{g}_{(n+1)}{\prime}}_{13}\\ {{g}_{(n+1)}{\prime}}_{23}\end{array}\\ \begin{array}{c}{{g}_{(n+1)}{\prime}}_{33}\\ {{g}_{(n+1)}{\prime}}_{43}\end{array}\end{array}\end{array}\right]$$

18 $${\overrightarrow{E}}_{n}=\left\{{\text{row}}_{i}\left[{G}_{n}^{{{\prime}}}\right]-{\text{row}}_{j}\left[{G}_{n}^{{{\prime}}}\right]| i,j\in \left\{\text{1,2},\text{3,4}\right\}, i\ne j\right\}$$

19 $${\overrightarrow{E}}_{(n+1)}=\left\{{\text{row}}_{i}\left[{G}_{(n+1)}^{{{\prime}}}\right]-{\text{row}}_{j}\left[{G}_{(n+1)}^{{{\prime}}}\right]| i,j\in \left\{\text{1,2},\text{3,4}\right\}, i\ne j\right\}$$

Subsequently, it computes the normal vectors of all potential separating planes between the rectangles by utilising the cross-product of their respective edges.

For each pair of edges, one from $\left[{G}_{n}^\prime\right]$ and one from $\left[{G}_{(n+1)}^\prime\right]$, calculate the cross-product to obtain the normal vector $\overrightarrow{\Psi }$ of the potential separating plane:

20 $$\overrightarrow{\Psi }=\left\{{{\overrightarrow{E}}_{n}}_{i}\times {{\overrightarrow{E}}_{(n+1)}}_{j} | i,j\in \left\{1, 2, 3, 4\right\}\right\}$$

For each normal vector, divide it by its magnitude to obtain a unit vector:

21 $$\widehat{\Psi }=\left\{\frac{{\psi }_{i}}{\Vert {\psi }_{i}\Vert } |{ \psi }_{i}\in \overrightarrow{\Psi }\right\}$$

The essence of the function lies in evaluating overlaps across all potential separating planes. To accomplish this, the algorithm projects the corners of both rectangles onto the current separating plane and checks for any overlap along that plane. If an overlap is detected, the function continues to examine the next plane. However, if a separating plane is found to be non-overlapping, the function returns a boolean variable ‘isIntersecting’ as false, accompanied by an empty ‘intersectionBox’.

For each normalized normal vector $\widehat{\psi }$

Project the corners of both rectangles ${G}_{n}^\prime$ and ${G}_{(n+1)}^\prime$, onto the separating plane defined by $\widehat{\psi }$:

22 $${P}_{n}=\left\{\widehat{\psi }.{\text{row}}_{i}\left[{G}_{n}^\prime\right] | i\in \left\{1, 2, 3, 4\right\}\right\}$$

23 $${P}_{(n+1)}=\left\{\widehat{\psi }.{\text{row}}_{i}\left[{G}_{(n+1)}^\prime\right] | i\in \left\{1, 2, 3, 4\right\}\right\}$$

When the function completes its loop without identifying a separating plane, it concludes that the rectangles intersect and returns ‘‘ as true. The ‘isIntersecting’ variable acts as an identifier for each rectangle, helping to exclude intersecting areas during the generation of the surface model. The rectangle that intersects with the highest number of other rectangles will be excluded, and the algorithm will reassess the remaining rectangles for intersections. This process continues until all intersections have been resolved. This function provides a reliable and efficient method for detecting intersections between 3D rectangular regions, making it a valuable tool for various applications, including computer graphics, physics simulations, and computational geometry.

24 $${\mathbb{G}}=\left\{\begin{array}{c}={G}_{n}^\prime, isIntersecting=False\\ \ne {G}_{n}^\prime, isIntersecting=True\end{array}|i\in \left\{1, 2, 3, \dots , n\right\}\right\}$$

### STL model of the plate

After the intersecting rectangles have been removed, the point cloud undergoes further processing to generate a surface model of the plate. The resulting surface model is devoid of any holes. To create holes, a Boolean operation is performed, and a cylindrical surface model of the hole is subtracted from the plate.

The surface model of the hole is constructed by defining circles along the z-axis at various heights and connecting the peripheral points of these circles to form the overall surface, as illustrated in the figure. A software application has been developed to generate a 3D surface model of the hole based on specified parameters, including diameter (φ), head diameter (Ø), height (Ħ), head height (ħ), and head angle (ß). This model is represented as a mesh composed of vertices and faces. The program employs mathematical functions and geometric operations to create the mesh, visualize the hole, and save the model in an ASCII STL file format.

Initially, the diameters and heights for various parts of the hole are defined using the input parameters. The centre points of different circles are then calculated along the Z-axis based on the height, head height, and head angle. Subsequently, a set of points on the periphery of each circle is computed using the parametric equation of a circle, determining the X, Y, and Z coordinates for each point.

The program creates the vertices and faces of the hole mesh by iterating through the circle points and triangulating them using geometric operations. This process establishes connectivity between adjacent circles, forming the hole’s surface. Furthermore, the ends of the hole are closed by connecting the first and last circles to their respective centre points using additional faces. The resulting mesh is used to subtract from the surface model of the plate to create the holes.

Before the Boolean operation, the surface model of the hole is translated and positioned at the appropriate location, as illustrated in Fig. 4(c). The surface model of the hole is aligned with the hole vector, which is the direction vector of the hole’s polar axis. Before executing the Boolean operation, it is necessary to examine the surface models for errors related to facet orientation. The zoomed-in area of Fig. 4(c) reveals that the normals of each facet point in the outward direction, indicating that an error-free STL model of the plate will be generated after the Boolean operation.

To perform a Boolean subtraction operation between a surface model of the plate and the hole, a modified version of the computer program developed by Eric Trudel (2023) [12] is employed, with some changes made to the core functionality. The code effectively handles surface Boolean operations for triangular mesh representations of the 3D shapes.

Initially, the input meshes undergo visualization and preprocessing to evaluate their quality and ensure water tightness. Following this, the algorithm computes the intersection curves between the two shapes, which results in the formation of intersection loops. Next, subsurfaces are created, during which the code identifies the specific Boolean operations associated with each subsurface, such as union, subtraction, or intersection. For the algorithm presented, modifications have been made to allow the code to save the STL file of the plate generated by the subtraction process.

**Fig. 8:**
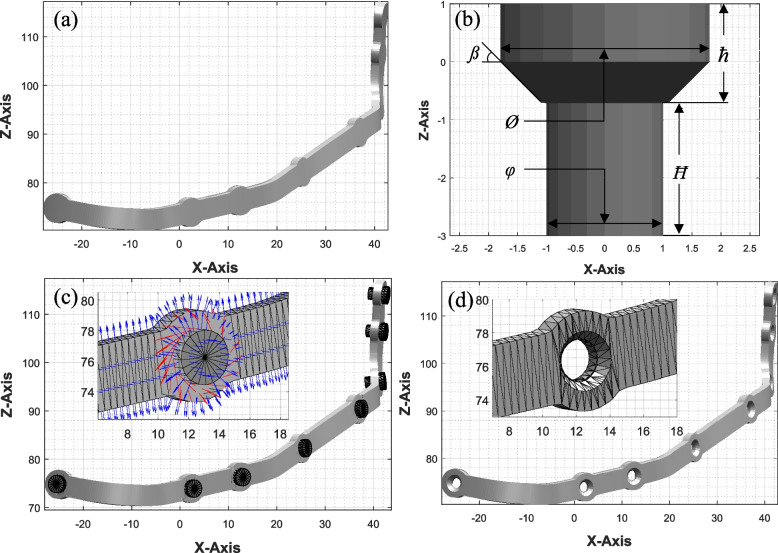
**a** Surface model of the plate without hole, **b** Surface model of the hole showing different parameters, **c** Surface model of different holes aligned with the hole axis and position, zoomed in area of (7,73) and (18,80) showing the different facets and their normal pointing in outwards direction. **d** STL model of the surgical plate, zoomed in area of (7,73) and (18,80) showing the countersunk hole for the screw fastener

The code generates the desired resulting shape of the plate as shown in Fig. 8(d), which is the subtraction of the surface model of the hole Fig. 8(b) from the surface model of the plate Fig. 8 (a). This outcome is then exported as an ASCII STL file, paving the way for further analysis or application. This approach ensures an efficient and accurate implementation of Boolean operations on 3D shapes, allowing for the creation of complex geometries.

After creating the CAD model of the PSI, the STL model is virtually assembled with the reconstructed part, as shown in the figure, to verify its functionality and accuracy. This is followed by FEA to ensure the structural strength of the plate and fabrication using additive manufacturing.

We simulate the structural strength of plates utilized in clenching tasks within a FEA framework. The boundary conditions for these simulations are derived from a thorough review of pertinent literature [18, 19], with modifications made to fit our specific context. Muscle forces are scaled based on the calculated tooth reaction force (occlusal force) and the pre-operative measured maximum occlusal force to reflect clenching accurately under post-operative conditions. By adjusting the magnitudes of these forces according to the patient’s preoperative maximum occlusal force, we can effectively simulate post-operative conditions while considering the expected significant reduction in bite force [9, 13] This methodology allows us to establish a reasonable margin for evaluating the plate’s potential structural failure, which is a crucial factor of safety.

### Boundary conditions

This research article employed a mechanistic model to calculate tooth responses based on muscle force vectors. According to the first principle of mechanics, the sum of all forces applied to the mandible and the sum of the moments created by these forces will equal zero. The reaction force at the point of resistance (both condyle and teeth) was computed. These resistance vectors included x, y, and z components, akin to the force vectors. Consequently, a total of nine unknowns needed to be determined from six equations. Solving these six equations with nine unknowns was not feasible. However, these equations could be resolved by considering several reasonable assumptions outlined by Nelson (1986) [20].

25 $$\sum F_x=0$$

26 $$\sum {F}_{y}=0$$

27 $$\sum {F}_{z}=0$$

28 $$\sum {M}_{x}=0$$

29 $$\sum {M}_{y}=0$$

30 $$\sum {M}_{y}=0$$

where:

*F*_*x*_, *F*_*y*_, and *F*_*z*_ are the force vector components in x, y, and z directions respectively

*M*_*x*_, *M*_*y*_, and *M*_*z*_ are the moments of various forces along in x, y, and z directions respectively

The orientation of the tooth resultant vector is defined in terms of α (lateral) and β (frontal) angles. Furthermore, it is assumed that the reaction vector of the left condyle forms a γ angle with the x-axis. This assumption can be expressed as:

31 $$\text{tan}\left(\alpha \right)=\frac{Tz}{Ty}$$

32 $$\text{tan}\left(\beta \right)=\frac{Tz}{Tx}$$

33 $$\text{tan}\left(\gamma \right)=\frac{CLz}{CLx}$$

where:

*T*_*x*_, *T*_*y*_, and *T*_*z*_ are the tooth reaction components in the x, y, and z directions respectively

*CLx* and *CLz* are reaction forces at the left condyle along the x and z axes

By solving the Eqs. (24–33) for the muscle force values listed in Table, and assuming values of α, β and γ are assumed 100°, 70° and 90° respectively three reaction vectors are calculated: one at the tooth and two at the condyles. The muscle forces are then scaled according to the tooth reaction force (*T*) obtained from the calculation and cleaning force 91N measured at the right molar before the patient’s surgery. These scaled muscle force values simulate the clenching task using FEA.

### FEA of the plate

**Fig. 9:**
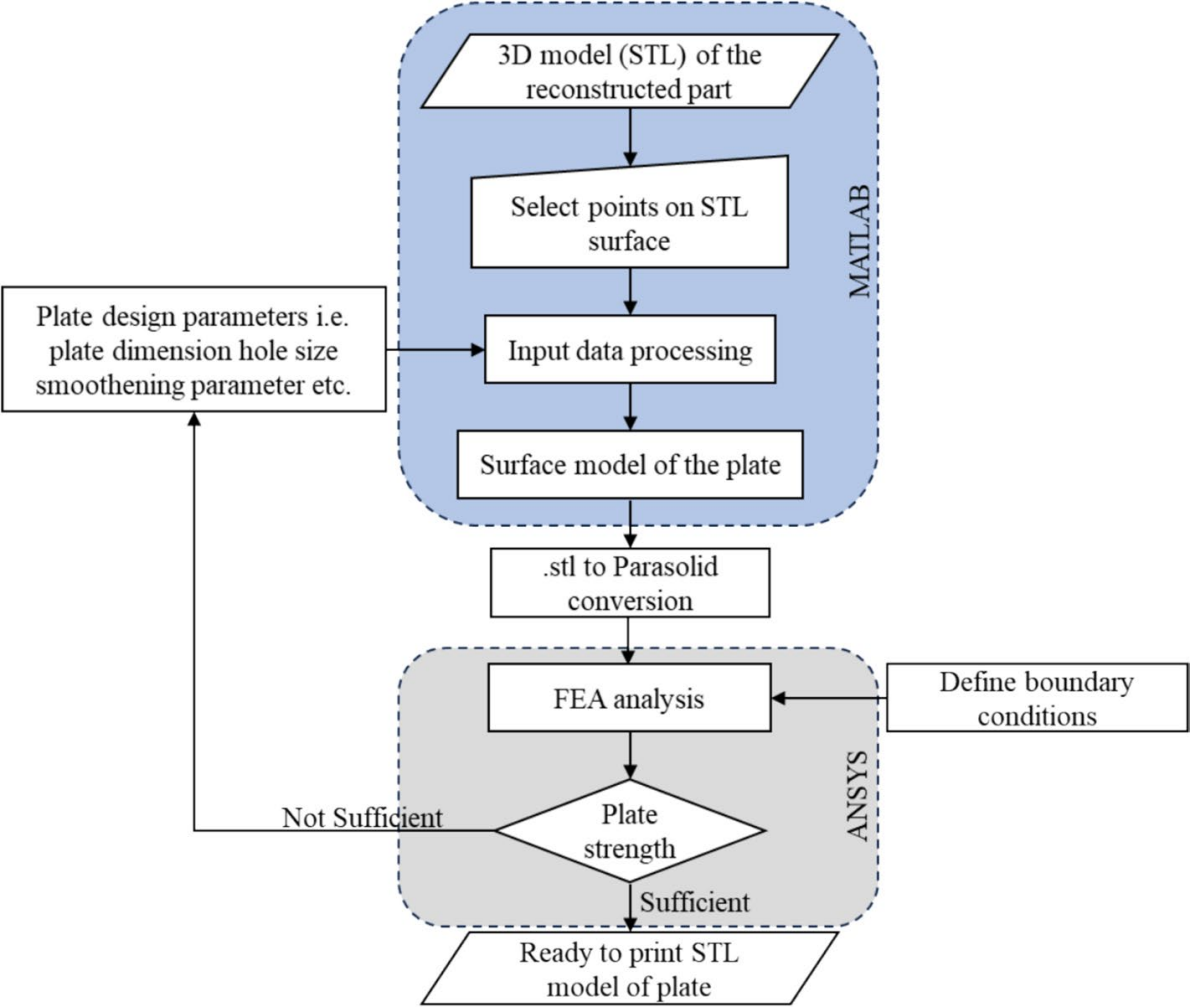
Process flow diagram

FEA using ANSYS is performed to evaluate and confirm the strength and functionality of the PSI designed with the proposed method. As depicted in the Fig. 9 The designed plate is being tested for its strength. If it fails the testing, the design parameters will need to be updated, followed by the redesign.

The STL model of the reconstructed part is processed in 3Matic to remove any spikes and to regenerate the triangular mesh of the STL file with a uniform density. This consistent triangulation enables the calculation of a watertight surface model, which can then be converted into a solid model suitable for FEA The PSI is assembled with the reconstructed model and imported into ANSYS Workbench for meshing. A ten-node tetrahedral mesh is utilized, with varying mesh sizes assigned to different assembly components: the bone, plate, and screw are designated mesh sizes of 3.0 mm, 1 mm, and 0.3 mm, respectively. Furthermore, a mesh convergence analysis is performed to ensure the accuracy of the model’s mesh density.

For computational work simplification, the mesh model of assembly is assumed to be homogeneous, isotropic and linearly elastic. PSI and screw were assigned with the material properties of Titanium alloy (Ti-6AL-7Nb) having Young’s modulus 105 GPa and Poisson’s ratio 0.36 [14]. The reconstructed mandible was modelled to be a dense cortical bone having Young’s modulus 13,700 MPa and Poisson’s ratio 0.3 based on considerations as indicated by Qimin et al. (2021) [15].

The imported assembly comprises various components, including a repositioned mandible, a reconstructed segment of the fibula free flap, a PSI, and screws to secure the physical assembly. In multibody FEA simulation, it is essential to define the contact between different mating parts to impose the correct mathematical conditions in computational models that replicate the real-world interaction of the respective bodies. In the study presented, frictional contact has been utilised at the interface of the screw with the bone and PSI. The coefficient of friction between the screw and the bone is set at 0.3, while the interface between the screw and the plate is defined as a ‘bonded’ interface. The interface between the plate and the bone is characterised as a frictionless bond, which only allows for a normal reaction and prevents the virtual model of the PSI and bone from intersecting with each other.

**Fig. 10:**
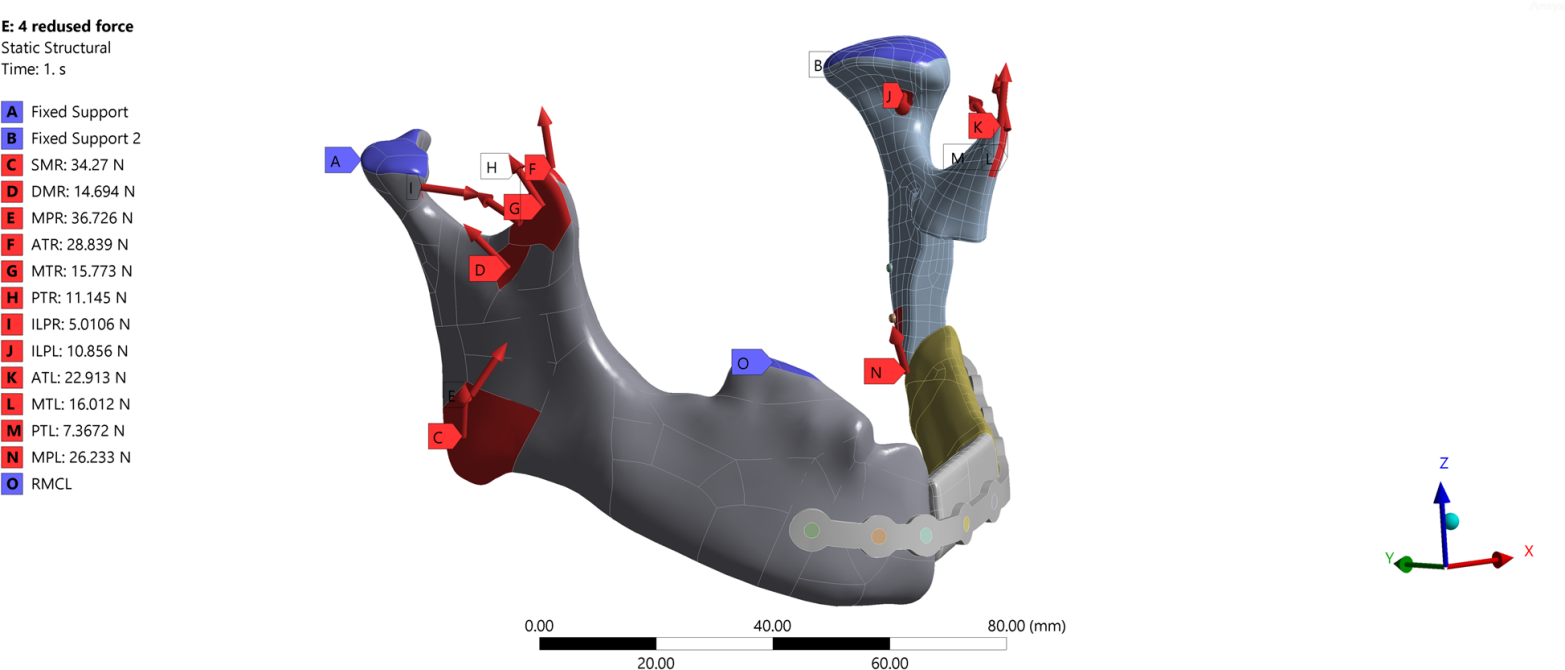
Muscle force direction and value included Superficial Masseter (SMR), Deep Masseter (DMR), Medial Pterygoid (MPR), Anterior Temporalis (ATR), Middle Temporalis (MTR), Posterior Temporalis (PTR), Inferior Lateral Pterygoid (ILPR) for the right side of the mandible and Medial Pterygoid (MPL), Anterior Temporalis (ATL), Middle Temporalis (MTL), Posterior Temporalis (PTL), Inferior Lateral Pterygoid (ILPL) for the left side of mandible, and RMCL showing the fixed support in normal direction of the molar teeth to simulate right molar clenching

Muscle forces to simulate right molar clenching are listed in Table 2. The force is in accordance with the literature [16–19]. Since the left superficial masseter and deep masseter were detached during the surgery, these force vectors are not considered for this simulation. For right molar clenching the condyles are fixed along x, y, and z directions and the right molar is supported as frictionless support that restricts the movement of the molar in the z + direction (as shown in Fig. 10) [16].

## Results

The results of the presented study can be divided into three parts: 1) Output of the developed algorithm, which delivers a CAD model of the PSI; 2) Reaction Force Determined from the Mechanistic Model; and 3) Results of the FEA.

**Fig. 11:**
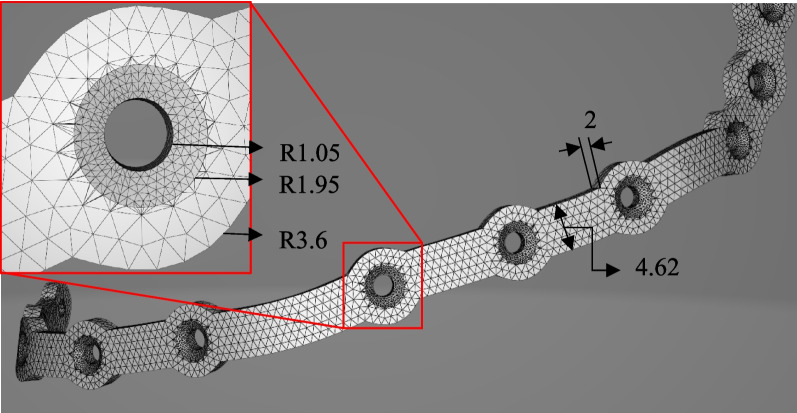
Generated STL file from the computer program, zoomed image of a hole showing adaptive tessellation near the hole boundary to minimize the volumetric error

1) Output of the developed algorithm: The proposed algorithm significantly improves efficiency and speed compared to traditional CAD model design methods. Defining the curves takes around one minute while processing the data and generating the CAD model, which requires an additional 40 to 60 s. This represents a considerable advancement over conventional methods. The generated STL model showcases high-quality (Fig. 11), exhibiting no errors such as holes, missing or overlapping facets. An accompanying figure illustrates the error-free STL model.

This innovative approach to CAD modeling has the potential to conserve time and resources while ensuring high-quality results. A statistical analysis of the triangles reveals that the model consists of 19,116 triangles. The minimum triangle size is 6.9559e-09 mm, the maximum is 0.44 mm, and the average is 0.11 mm.

2) Reaction Force Determined from the Mechanistic Model: Upon analyzing the mechanistic model for muscle forces as outlined in Table 1, the resultant tooth reaction vector is calculated to be 365.13 N. Specifically, the reactions at the left and right condyles measure 125.02 N and 336.47 N, respectively. The ratio of the total joint, or condylar force, to the total tooth reaction force is 1.26. It is posited that the resistance forces at the joints are residual effects of the useful force generated at the teeth, arising solely as a stabilizing influence on the system. Accordingly, the ratio of these forces reflects an aspect of the system’s efficiency in terms of force distribution. The scaled muscle forces, derived from the calculated tooth reaction and bite force, are detailed in Table 2.

**Table 1:** Muscle force value is used to solve the mechanistic model

Side	Muscle Name	X (N)	Y (N)	Z (N)
Right	Superficial Masseter (SMR)	−28.38	57.44	121.19
	Deep Masseter (DMR)	−32.08	−21.03	44.53
	Medial Pterygoid (MPR)	71.36	54.77	116.14
	Anterior Temporalis (ATR)	−17.19	5.07	113.96
	Middle Temporalis (MTR)	−14.01	−31.55	52.81
	Posterior Temporalis (PTR)	−9.28	−38.14	21.14
	Inferior Lateral Pterygoid (ILPR)	12.63	15.17	−3.49
Left	Medial Pterygoid (MPL)	−50.97	39.12	82.96
	Anterior Temporalis (ATL)	13.65	4.03	90.54
	Middle Temporalis (MTL)	14.22	−32.03	53.61
	Posterior Temporalis (PTL)	6.13	−25.21	13.98
	Inferior Lateral Pterygoid (ILPL)	−27.35	32.87	−7.56

**Table 2:** Scaled muscle force value used to simulate right molar clenching

Side	Muscle Name	X (N)	Y (N)	Z (N)
Right	Superficial Masseter (SMR)	−7.09	14.36	30.30
	Deep Masseter (DMR)	−8.02	−5.26	11.13
	Medial Pterygoid (MPR)	17.84	13.69	29.04
	Anterior Temporalis (ATR)	−4.30	1.27	28.49
	Middle Temporalis (MTR)	−3.50	−7.89	13.20
	Posterior Temporalis (PTR)	−2.32	−9.53	5.29
	Inferior Lateral Pterygoid (ILPR)	3.16	3.79	−0.87
Left	Medial Pterygoid (MPL)	−12.74	9.78	20.74
	Anterior Temporalis (ATL)	3.41	1.01	22.64
	Middle Temporalis (MTL)	3.55	−8.01	13.40
	Posterior Temporalis (PTL)	1.53	−6.30	3.49
	Inferior Lateral Pterygoid (ILPL)	−6.84	8.22	−1.89

The data and the figure of the forces on the right and left sides show that the muscles on the right side exert more force than those on the left side.

**Fig. 12:**
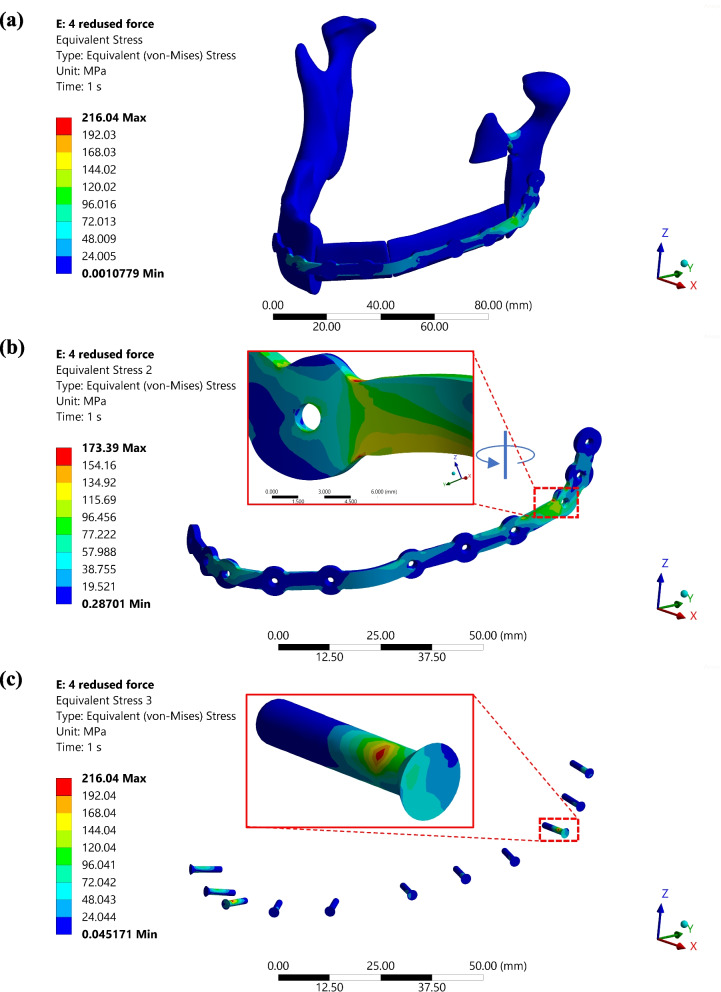
**a** Equivalent (von-Mises) stress graph for the reconstructed mandible assembly, **b** Equivalent stress distribution in PSI, zoomed image showing the high stress concentration zone for plate. **c** Equivalent stress distribution in different screws, the zoom image shows the screw which faces the maximum stress

3) Results of the FEA: The maximum stress in the plate registers at 173.39 MPa (see Fig. 12(b)). In comparison, the 9th screw, situated near the junction of the reconstructed and reaction components of the mandible, displays a maximum von Mises stress of 216.04 MPa (Fig. 12(a)), which is below the ultimate yield strength of titanium alloy, i.e. > 800 MPa [20]. However, these stress levels are at the threshold of grade-I pure titanium yield strength, which is 170 MPa [21].

**Fig. 13:**
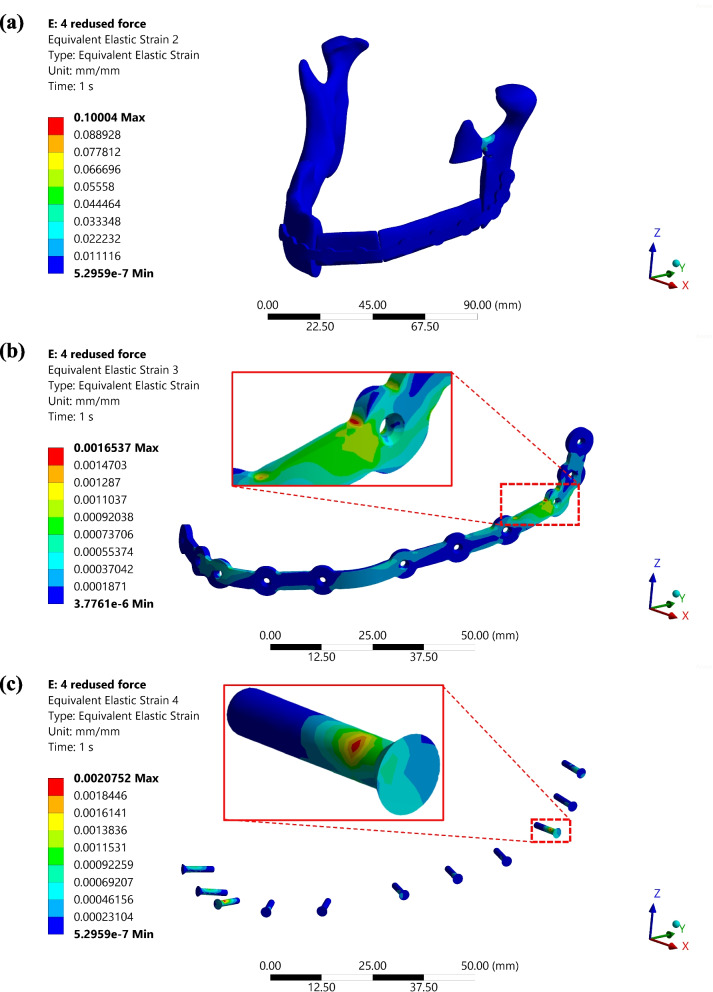
**a** Equivalent elastic strain graph for the reconstructed mandible assembly, **b** Equivalent elastic strain distribution in PSI, zoomed image showing the high-strain rate zone for the plate. **c** Equivalent elastic strain distribution in different screws, the zoom image shows the screw which faces the maximum strain

The strain distribution in the mandibular notch shows a value of 0.1, which can be attributed to structural changes in the mandible rather than having any significant impact on the plate. As indicated in the highlighted area of Fig. 13(b), the plate displays an elastic strain of 0.001. Simultaneously, the screw positioned at this location undergoes the maximum strain of 0.002, resulting from the junction between the left part of the mandible and the reconstructed bone. This screw plays a crucial role in load transfer between the plate and the mandible.

## Discussion

The algorithm developed for CAD modelling of maxillofacial implants automatically generates the CAD model based on defined design parameters, such as the dimensions of the plate and the smoothness factor. This algorithmic approach facilitates a more efficient and flexible design process than conventional methods, which involve time-consuming manual CAD modelling and editing of STL files. Although the specific design steps for the plate are not detailed in the literature, they are typically created by manipulating the bone’s STL file or primitive geometries through Boolean operations. This process is laborious and requires specific engineering skills, making it challenging for surgeons to execute. Another common issue with STL manipulation is the potential for errors that must be addressed in preprocessing. However, the developed algorithm overcomes this problem by generating error-free STL files with adaptive meshing, ensuring accurate details and avoiding volumetric errors related to CAD modelling. In contrast to conventional methods, the presented algorithm does not require specialised knowledge of CAD modelling to design PSIs, making it a more accessible and efficient solution.

The sweep-based surface modelling approach creates a smooth PSI (plate) CAD model, eliminating sharp edges or vertices that could cause stress concentration. The FEA demonstrates that the designed plate can withstand forces under simulated conditions for right molar clenching. However, it is essential to consider the variability in clenching forces among individuals, as using the same forces from the literature may not be suitable for everyone. Furthermore, post-surgery conditions may vary; some muscles could be detached from the mandible during surgery and may not participate in bite actions, thus not applying forces. Such post-operative conditions should also be addressed.

To tackle this issue, the patient’s maximum bite force at the right molar 46 was measured, and the boundary conditions [17, 18] for the FEA were adjusted accordingly.

A more accurate and personalised representation of the patient’s bite force is achieved by applying the adjusted force value as the boundary condition for the FEA analysis. This ensures that the implant plate design is better suited to the individual’s needs, considering pre-existing conditions and potential post-operative changes. It is obvious that there are always certain changes in the bite fore after the surgery due to muscle loss and other causes, and it is significantly reduced [10–14].

The yield strength of 3D printed titanium alloy Ti-6Al-7Nb can vary depending on the specific additive manufacturing process used, such as Selective Laser Melting (SLM) or Electron Beam Melting (EBM), as well as the processing parameters and post-processing treatments. Generally, the yield strength of 3D-printed Ti-6Al-7Nb falls within the 800–1100 MPa range [22, 23]. If the plate is fabricated using this alloy, it will operate with a safety factor 4.5. Conversely, if the plate is considered for fabrication using titanium grade 1, it may exhibit some yielding at the specified force value. However, in practical scenarios, there is typically a reduction in bite force after surgery, which allows for some margin regarding the plate’s yielding until the joint’s healing of bone segments occurs and becomes the primary weight-bearing structure.

The highest levels of stress are observed near the 9th screw location on the plate and within the screw itself, indicating that the majority of the load is transferred to the plate through this screw. Variations in cross-section and geometric curvature significantly influence the flow of stress, leading to increased stress concentration in the neck area of both the bolt and the plate, as illustrated in Fig. 12(b). While the plate’s design could be enhanced to manage this stress better, it does not reach the ultimate yield point, thus it can be deemed a safe design.

The algorithm created for plate design was evaluated across five cases. When applying this algorithm, it is crucial to define specific parameters that affect the plate’s smoothness and structure, such as the allowable error in cubic smoothing splines, along with the plate’s thickness and width. The smoothness of the curve pertains to the moving average of the points. Therefore, it is advisable to establish the curves before the placement of the holes, ensuring that the holes remain accurately positioned. Furthermore, the algorithm autonomously identifies the locations of the plate’s endpoints, eliminating the need to define the first and last hole manually. This feature enhances the design process, improving both the efficiency and accuracy of the plate design algorithm.

The current model allows design for both single-segment and multi-segment reconstructions, which cover most of the osseous reconstruction scenarios. However, all the screw holes need to be in a line as in single-barrel fibula reconstruction. It exhibits some limitations in modeling the complex reconstruction plate where multiple bars might be connected to a single screw hole. The algorithm presented here focuses on the design of PSI specifically for single-barrel reconstruction. Currently, we are working closely to improve it for more intricate designs like those described in our recent publication [24]. Once the plate is designed, it must be inspected for its functionality and geometry. Since design and modeling depend on input parameters, there are instances where fine-tuning is necessary to achieve the optimal design for a specific case. However, the algorithm generally generates the most suitable model for any reconstruction scenario.

Developing models that involve intricate biological interactions requires effective management and control of numerous variables. While an ideal model should account for all relevant factors, certain assumptions must be made to identify the most critical variables and their incorporation. One such model is the mechanistic model presented, which assumes no relative movement between the various parts of the reconstructed mandible. To simplify the analysis of force variables, resistance force distributions are calculated by designating the left condyle frontal angle of resistance. In the static analysis, the positions of tooth and joint contact for resistance forces are treated as single points rather than surfaces or multiple points.

One limitation of the FEA model in this study is that it exclusively analyzes the right molar clenching task. This constraint arises from the anatomy of the reconstructed mandible, which only permits the simulation of right molar clenching due to the absence of left molar teeth and the detachment of the masseter muscles on the left side. Nevertheless, given that the safety factor for titanium alloy exceeds 4, it can be concluded that the plate will perform reliably without yielding or fracturing. Additionally, increasing the plate’s width could enhance its ability to manage stress concentration.

## Conclusion

The novel algorithm presented in this article has the potential to fundamentally transform the field of patient-specific implant design by streamlining the traditionally intricate and time-consuming CAD process. It enables surgeons to create patient-specific implants with enhanced ease, efficiency, and reliability. This algorithm is poised for widespread adoption among clinicians, addressing the challenges inherent in preoperative virtual surgical planning (VSP). Moreover, this innovative approach marks a significant advancement toward AI-assisted VSP, utilizing computational capabilities to design the optimal implant automatically. The following conclusions can be drawn from this study:

1. Swapping a rectangle along a curve with adopting the curvature could be used to design PSI for jaw reconstruction.
2. The cubic smoothing spline results in a smooth curvature of the plate that helps to reduce stress concentration at sharp edges and corners.
3. The method described significantly decreased the time needed to design a patient-specific implant.
4. Mechanistic model can be used to scale the standard (reported in the literature) muscle forces, as per the patient bite force.
5. The proposed approach can result in PSI designs that have sufficient strength.

## Data Availability

No datasets were generated or analysed during the current study.
